# Benefits and Complications of Long-Term Low-Dose Glucocorticoids in Rheumatoid Arthritis: A Traditional Review

**DOI:** 10.7759/cureus.117932

**Published:** 2026-10-14

**Authors:** Malk Al-Shudifat, Rahul Satheesh, Wodwentzky Lefranc, Miguel Rivas-Perez, Malvika Sawai, Laraib Iftikhar

**Affiliations:** 1 Internal Medicine, Jordanian Ministry of Health, Mafraq, JOR; 2 Medicine, Ras Al-Khaimah Medical and Health Sciences University, Ras Al Khaimah, ARE; 3 Internal Medicine, Ascension Health, Milwaukee, USA; 4 Department of Surgery, Universidad de Oriente, Bolivar, VEN; 5 Medicine, Himalayan Institute of Medical Sciences, Dehradun, IND; 6 Emergency Medicine, California Institute of Behavioral Neurosciences and Psychology, Fairfield, USA

**Keywords:** cardiovascular risk, complications, disease activity, efficacy, glucocorticoids, glucocorticoid withdrawal, infection risk, low-dose prednisone, osteoporosis, rheumatoid arthritis

## Abstract

Long-term low-dose glucocorticoid therapy remains widely used in rheumatoid arthritis (RA), despite concerns regarding cumulative toxicity. This traditional review evaluates the clinical benefits and complications of oral glucocorticoids at doses of ≤7.5 mg/day prednisone equivalent in adults with RA, with particular attention to disease control, bone health, cardiovascular outcomes, infection, and glucocorticoid withdrawal. A literature search of PubMed Advanced, PubMed Central, and Google Scholar conducted from July 1 to October 9, 2026, identified 56 potentially relevant studies, of which 36 were selected for qualitative synthesis. Evidence from randomized controlled trials, observational cohorts, systematic reviews, meta-analyses, and clinical guidelines was evaluated.

Low-dose glucocorticoids provide measurable clinical benefits, including reduced disease activity, prevention of disease flares, and slowing of radiographic joint damage. However, these benefits must be balanced against cumulative adverse effects. Infection was the most consistently demonstrated complication, with observational evidence showing an increased risk even at doses of ≤5 mg/day. Evidence regarding skeletal toxicity was mixed: observational studies associated low-dose glucocorticoids with reduced bone mineral density (BMD) and increased fracture risk, whereas randomized trial data suggested less pronounced effects when inflammation was controlled and bone-protective therapy was used. Cardiovascular risk also remained uncertain at very low doses and was influenced by baseline cardiovascular risk and cumulative exposure. Early initiation of effective biologic or targeted synthetic disease-modifying antirheumatic drugs (DMARDs) was associated with greater success in glucocorticoid discontinuation, while tapering could increase the risk of disease flare in patients with established low disease activity.

Overall, low-dose glucocorticoids retain clinically meaningful efficacy in RA, but long-term use is associated with important cumulative risks, particularly infection. When continued therapy is necessary, the minimum effective dose should be used with regular reassessment and appropriate monitoring and preventive measures. When disease control permits, gradual tapering and steroid-sparing therapy should be prioritized.

## Introduction and background

Rheumatoid arthritis (RA) is characterized by persistent inflammation in joints that leads to damage in the surrounding cartilage and bone [1]. The disease affects approximately 0.5% to 1.0% of the global population [2], with incidence rates rising progressively after age 40 [3]. Epidemiological data indicates a prevalence of approximately five per 1,000 individuals [4], with comprehensive systematic analyses tracking global, regional, and national burden trends over recent decades [5], and population-based systematic reviews further confirming these prevalence patterns [6]. Both the disease and its treatment cause multiple comorbidities, such as an increased incidence of cardiovascular disease, diabetes mellitus, and osteoporosis and fragility fractures [7]. RA also increases the risk of hospitalized infections. These factors make treatment decisions more complex.

Glucocorticoids (GCs), especially prednisone and prednisolone, have been used in the treatment of RA for a long time, including for more than six months, due to their anti-inflammatory and immunosuppressive effects. Current international guidelines recommend combining synthetic or biologic disease-modifying antirheumatic drugs (DMARDs) with short-term GC bridging therapy while aiming for a swift taper [8], whereas other major clinical guidelines provide conditional recommendations against routine long-term GC use [9]. Modern consensus frameworks also establish clear definitions for short-term, long-term, and chronic GC exposures alongside practical clinical guidance [10]. Furthermore, the timing of biologic or targeted synthetic (b/tsDMARD) initiation plays a critical role in facilitating successful GC withdrawal and minimizing cumulative exposure [11]. Low doses of GCs ≤7.5 mg for long periods of time raise concerns about possible toxicities and side effects, especially in older adults. Reportedly, 30% to 60% of patients with RA remain on long-term GCs, especially at low doses [12]. Clinicians struggle to balance the undeniable clinical efficacy of these low doses against the cumulative, insidious systemic toxicities that develop over prolonged periods.

It is well established that GCs are associated with several adverse events, most of them related to dose and time of exposure [13]. Observational studies, meta-analyses, and randomized clinical trials have reported dose-dependent side effects, including bone mineral density (BMD) loss, osteoporosis, fractures, cardiovascular events, diabetes mellitus, and infections. GC-induced osteoporosis (GIOP) is a particularly important complication of prolonged GC exposure and is characterized by increased fracture risk associated with GC therapy. To help optimize safety, an expert task force has established dose-stratified risk frameworks defining acceptable levels of harm across specific daily dosage thresholds [14]. Even low-dose GCs for prolonged periods of time can be harmful. To date, studies remain inconsistent regarding the magnitude of harm of low-dose GCs, when these side effects start to appear, after how long, and what combination of drugs increases the risk of side effects. Furthermore, each of these questions warrants further investigation.

Although the efficacy of low-dose GCs in reducing disease activity and slowing radiographic progression has been demonstrated, uncertainty remains regarding their long-term safety, particularly with respect to GIOP and fragility fractures, cardiovascular outcomes, infections, metabolic complications, and the clinical consequences of continued treatment versus tapering. Evidence is also heterogeneous with respect to patient age, disease duration, GC dose, duration of exposure, and concomitant DMARD therapy. Therefore, this traditional narrative review summarizes contemporary evidence regarding the benefits and complications of long-term low-dose oral GC therapy in adults with RA, with particular attention to efficacy, bone health, cardiovascular outcomes, infection risk, and GC tapering. 

## Review

Methodology and search strategy

Study Design and Search Framework

We designed this traditional narrative review to weigh the clinical benefits of low-dose GC therapy against its long-term risks in adults with RA. Using the Patient, Intervention, Comparison, and Outcome (PICO) framework, we focused on adult patients receiving a baseline daily dose of ≤7.5 mg/day prednisone-equivalent GC. For dose conversion, prednisone 5 mg is considered approximately equivalent to prednisolone 5 mg and methylprednisolone 4 mg; thus, the review's upper threshold of 7.5 mg/day prednisone equivalent corresponds approximately to 7.5 mg/day prednisolone or 6 mg/day methylprednisolone. Because dosing definitions vary across published trials, we also included studies examining strict cutoffs of ≤5 mg/day or narrow ranges between 2.5 and 7.5 mg/day. The primary focus of this review was long-term or chronic GC use, particularly treatment extending beyond six months. Studies of shorter duration were included when they provided relevant evidence regarding efficacy, safety, bone health, infection, or GC withdrawal and were interpreted as contextual rather than as direct evidence of long-term exposure. Key outcomes centered on disease activity and radiographic progression, BMD, GIOP and fragility fractures, cardiovascular outcomes, serious or hospitalized infections, metabolic complications including hyperglycemia, and outcomes associated with GC tapering or withdrawal.

Information Sources and Search Execution

Between July 1 and October 9, 2026, we searched PubMed Advanced, PubMed Central (PMC), and Google Scholar using keywords across four core themes: rheumatoid arthritis, glucocorticoids, low-dose regimens, and clinical outcomes. To ensure we did not miss valuable papers that looked only at safety or only at efficacy, we avoided requiring both terms in a single query. Our PubMed Advanced search string was: (rheumatoid arthritis OR RA) AND (prednisone OR prednisolone OR methylprednisolone OR glucocorticoid OR corticosteroid) AND (low dose OR low-dose OR low dosage) AND (complications OR adverse events OR glucocorticoid-induced osteoporosis OR toxicity OR safety OR efficacy OR outcome). For PMC and Google Scholar, we ran phrase searches using rheumatoid arthritis AND prednisone AND low-dose, reviewing the top 150 PMC results and the top 110 Google Scholar results ranked by relevance. We also manually checked the reference lists of included articles to catch any additional relevant studies.

Eligibility Criteria

We included peer-reviewed, English-language studies on human adults with RA published between 2016 and 2026 that evaluated low-dose oral GCs, including prednisone, prednisolone, or methylprednisolone, and were accessible in full text through open access or institutional access. Original clinical trials, observational cohorts, and meta-analyses provided our primary data for RA-specific efficacy. Clinical guidelines, narrative reviews, editorials, and studies covering mixed inflammatory populations (such as RA overlapping with systemic lupus erythematosus) were excluded from our primary efficacy evaluation. However, we drew on them selectively as background context when discussing broader steroid toxicity mechanisms. We excluded studies evaluating systemic GC regimens above the prespecified low-dose threshold of >7.5 mg/day prednisone equivalent when the exposure was not separately analyzable, intravenous pulse therapy or other very-high-dose regimens outside the scope of this review, animal studies, and non-English-language studies.

Study Selection and Yield

Our initial database search and reference check turned up 56 potential papers. After removing duplicates and reviewing full texts against our criteria, a total of 36 publications were included in the qualitative synthesis. These comprised primary clinical studies, observational cohorts, secondary analyses, systematic reviews and meta-analyses, clinical practice guidelines, consensus statements, narrative reviews, and one study protocol. We excluded the remaining 20 papers for specific reasons: a focus on high-dose or pulse therapy (n = 6), lack of separate RA data in mixed-disease cohorts (n = 5), duplicate cohort reporting in long-term extension trials (n = 6), and incomplete outcome data (n = 3).

Results and evidence synthesis

Primary clinical, observational, and secondary clinical evidence concerning the efficacy, safety, and steroid-sparing effects of systemic GC therapy in RA is summarized in Table 1 [1,7,11,12,15-27].

**Table 1:** Primary, observational, and secondary clinical evidence on systemic glucocorticoid therapy in rheumatoid arthritis RA: rheumatoid arthritis, TNF: tumor necrosis factor, GC: glucocorticoid, GLORIA: Glucocorticoid Low-dose in Rheumatoid Arthritis, b/tsDMARD: biologic or targeted synthetic disease-modifying antirheumatic drug, DMARD: disease-modifying antirheumatic drug, HR: hazard ratio, CVD: cardiovascular disease, DAS28: Disease Activity Score using 28 joint counts, BMD: bone mineral density, CPRD: Clinical Practice Research Datalink, NORD-STAR: Nordic Trial of Active Therapies for Early Rheumatoid Arthritis, PCSK9: proprotein convertase subtilisin/kexin type 9, DAS28-ESR: Disease Activity Score using 28 joint counts and erythrocyte sedimentation rate

Reference	Study design	Population	Sample size	Age	GC intervention/exposure	Comparator	Follow-up	Principal outcome	Principal finding	Main limitation
[1]	Retrospective cohort	RA patients initiating TNF inhibitor therapy	40933	Mean 53 years	Oral GC, ≤7.5 mg/day or >7.5 mg/day	No GC	Up to 2 years	Hospitalized infectious events	High-dose GC incidence: 13.3/100 person-years	Residual confounding
[7]	GLORIA trial protocol	RA with inadequate disease control	800 planned	≥65 years	Prednisolone 5 mg/day	Placebo	2 years	DAS28	Primary efficacy outcome prespecified	Protocol; no outcome data
[11]	Prospective cohort	RA initiating first b/tsDMARD	229	Mean 57 years	Very-low-dose GC, <5 mg/day	No GC	3 years	GC discontinuation	Permanent GC discontinuation: 23%	Single-center observational design
[12]	Retrospective cohort	RA receiving stable DMARD therapy	172,041 Medicare patients	Adults	GC ≤5 mg/day	No GC	1 year	Hospitalized infection	Infection incidence: 11.0% with ≤5 mg/day	Claims-based exposure misclassification
[15]	Population-based cohort	Six immune-mediated inflammatory diseases	87794	Mean 56 years	Oral prednisolone-equivalent GC	No GC	Median 5 years	Cardiovascular disease	HR for CVD with <5 mg/day: 1.74	Mixed disease population
[16]	Retrospective cohort	Newly diagnosed inflammatory/immune-mediated diseases	403337	18-64 years	Systemic GC	No GC exposure	Up to 8 years	Osteoporotic fracture	Fracture incidence with <5 mg/day: 9.33/1,000 person-years	Mixed disease population
[17]	Completed GLORIA randomized, double-blind, placebo-controlled trial	Established active RA	451	Mean 72 years	Prednisolone 5 mg/day	Placebo	2 years	DAS28	Between-group DAS28 difference: 0.37 points	Elderly population
[18]	Double-blind randomized controlled trial	Active early RA	395 randomized	Adults	Prednisolone 60 mg/day or 10 mg/day tapered to 0	Placebo	52 weeks	Radiographic progression	High-dose prednisolone showed no superiority over placebo	Physician-directed therapy after week 12
[19]	Secondary analysis of randomized trial	Active RA aged ≥65 years	449	≥65 years	Prednisolone 5 mg/day	Placebo	2 years	Body weight	Between-group weight difference: +1.3 kg	Secondary analysis
[20]	RA registry interim analysis	RA patients receiving low-dose GC	425	Adults	Prednisolone-equivalent 2.5–7.5 mg/day	No GC	Cross-sectional/interim	Lumbar-spine BMD	L1–L4 BMD difference: −0.042 g/cm²	Nonrandomized design
[21]	Longitudinal cohort	Women with inflammatory rheumatic musculoskeletal disease	884 women	Adult women	Prednisone-equivalent GC	Matched controls	Up to 6 years	Femoral-neck BMD	BMD change with ≥5 mg/day: −4.26%	Not RA-specific
[22]	Retrospective cohort	RA receiving stable DMARD therapy	135,583 Medicare patients	Adults	Oral GC by daily dose	No GC	1 year	Composite cardiovascular events	Predicted 1-year incidence with ≤5 mg/day: 1.4%	No significant association in CDM cohort
[23]	Population-based cohort	RA patients	12233	Adults	Time-varying prednisolone dose	No GC	Mean 8.7 years	Major adverse cardiovascular events	HR with ≥5 mg/day: 2.02	Observational residual confounding
[24]	Prospective cohort	Early RA	608	Mean 47.5 years	Low-dose GC	No/low cumulative exposure	10 years	Severe composite outcomes	Highest cumulative exposure group: 24.3% primary outcome	Observational cohort
[25]	Retrospective cohort	RA patients in UK CPRD	Not specified in primary abstract	Adults	Oral GC	No GC	Longitudinal	All-cause mortality	Current GC use HR: 1.77	Confounding by disease severity
[26]	Secondary biomarker analysis of randomized trial	Early RA	NORD-STAR participants	Adults	Systemic GC treatment	GC-sparing treatment strategy	24 weeks	PCSK9 concentration	GC treatment was associated with increased PCSK9	Surrogate biomarker outcome
[27]	Double-blind randomized controlled trial	RA with low disease activity on tocilizumab	259	Adults	Prednisone 5 mg/day	Prednisone taper to 0 mg/day	24 weeks	DAS28-ESR	Between-group DAS28 difference: 0.61 points	Conducted in patients receiving tocilizumab

Systematic reviews, meta-analyses, clinical practice guidelines, consensus statements, and contextual literature addressing GC-related adverse effects, tapering, bone health, and treatment recommendations are summarized in Table 2 [2-6,8-10,13,14,28-36].

**Table 2:** Systematic reviews, guidelines, consensus statements, and contextual evidence on glucocorticoid use in rheumatoid arthritis RA: rheumatoid arthritis, GC: glucocorticoid, DMARD: disease-modifying antirheumatic drug, GBD: Global Burden of Disease, EULAR: European Alliance of Associations for Rheumatology, csDMARD: conventional synthetic disease-modifying antirheumatic drug, ACR: American College of Rheumatology, SLE: systemic lupus erythematosus, HR: hazard ratio, BMD: bone mineral density, IPD: individual participant data

Reference	Study type	Population	Sample size	Age	GC exposure/intervention	Comparator	Follow-up	Principal outcome	Principal finding	Main limitation
[2]	Narrative review	Patients with RA	Not applicable	Adults	Systemic GC mechanisms	Targeted DMARD therapy	Not applicable	Inflammatory mechanisms	GCs suppress inflammatory pathways involved in RA	Narrative review
[3]	Narrative clinical review	Patients with RA	Not applicable	Adults	GC bridging within treat-to-target therapy	DMARD-based treatment	Not applicable	Treatment strategy	GCs are generally used as temporary adjunctive therapy	No independent quantitative analysis
[4]	Narrative clinical review	Patients with RA	Not applicable	Adults	GC bridging during DMARD initiation	DMARD therapy alone	Not applicable	Disease control	Early DMARD treatment reduces reliance on prolonged GC therapy	Broad clinical review
[5]	Global epidemiological analysis	Global RA population	Global population	All ages	Not applicable	Not applicable	1990–2017	RA burden	Age-standardized point prevalence: 246.6/100,000	Dependent on GBD modeling
[6]	Systematic review	Population-based RA studies	57 studies	All ages	Not applicable	Not applicable	Variable	RA prevalence	Global RA prevalence: 0.46%	Geographic heterogeneity
[8]	EULAR clinical practice recommendations	Adults with RA	Not applicable	Adults	Short-term GC bridging	csDMARD strategy without prolonged GC	Not fixed	GC duration	GCs should be tapered and discontinued as rapidly as clinically feasible	Recommendation depends on clinical context
[9]	ACR clinical practice guideline	Adults with RA	Not applicable	Adults	Short-term or long-term GC use	DMARD-based strategy	Not fixed	Treatment recommendation	ACR recommends avoiding long-term GC initiation	Conditional evidence for some recommendations
[10]	Expert commentary	Patients with RA receiving low-dose GC	Not applicable	Adults	Low-dose and very-low-dose GC	Dose concepts	Not applicable	GC terminology	Distinguishes low-dose from very-low-dose exposure	Expert commentary
[13]	Expert clinical review/debate	RA with low disease activity	Not applicable	Adults	Low-dose maintenance GC	GC cessation	Not applicable	Benefit–harm balance	Continued GC may maintain disease control in selected patients	Conceptual synthesis
[14]	EULAR task-force consensus	Patients requiring long-term GC	Not applicable	Adults	Prednisone-equivalent dose stratification	Dose-free reference	Not applicable	Acceptable harm	≤5 mg/day was identified as a lower-harm dose range in the consensus framework	Consensus-based
[28]	Narrative/mechanistic review	Patients with autoimmune rheumatic diseases	Not applicable	Adults	Systemic GC exposure	No fixed comparator	Not applicable	Glucose metabolism	GCs increase hepatic glucose production and insulin resistance	No quantitative pooled estimate
[29]	Systematic review	Chronic RA and SLE populations	10 included studies	Adults	Prolonged GC therapy and withdrawal	Tapering/withdrawal strategies	Variable	Withdrawal effects	Evidence supports gradual GC withdrawal when clinically feasible	Heterogeneous evidence
[30]	Systematic review and meta-analysis	RA and SLE with low disease activity/remission	472 participants	Adults	Continued GC ≤7.5 mg/day	GC tapering	24–104 weeks	Disease flare	Tapering HR for flare: 3.41	Very low certainty of evidence
[31]	Systematic literature review	Early and established RA	Multiple studies	Adults	GC bridging and maintenance	DMARD strategies	Variable	Efficacy and safety	Supports efficacy of GC bridging while documenting dose- and duration-related toxicity	Heterogeneous evidence
[32]	Systematic review and meta-analysis	RA populations	46,711 RA patients	Adults	GC exposure	RA controls	Variable	Bone mineral density	Lumbar-spine BMD difference: −0.038 g/cm²	Predominantly observational evidence
[33]	Systematic literature review and IPD meta-analysis	Early and established RA	1,112 participants with IPD	Adults	GC ≤7.5 mg/day	Placebo/standard care	2 years	Lumbar-spine BMD	BMD difference: −0.021 g/cm²	Few fracture events
[34]	ACR clinical practice guideline	Adults receiving chronic GC therapy	Not applicable	Adults	≥2.5 mg/day for >3 months	Risk-based osteoporosis management	Not fixed	Fracture prevention	Pharmacological treatment is strongly recommended for patients at high or very high fracture risk	Recommendations vary by fracture risk
[35]	Narrative review	RA receiving DMARDs and/or GC	Not applicable	Adults	GC with DMARD/biologic therapy	Lower/no GC exposure	Not applicable	Serious infection	Infection risk increases with GC exposure	Narrative synthesis
[36]	Narrative/mechanistic review	RA patients receiving GC	Not applicable	Adults	Systemic GC exposure	No fixed comparator	Not applicable	Bone metabolism	GCs reduce osteoblast activity and increase skeletal fragility	Mechanistic evidence

Studies incorporating mixed immune-mediated inflammatory conditions or combined cohorts, such as RA combined with systemic lupus erythematosus (SLE) or broader rheumatic diseases, are included strictly as supportive evidence to contextualize systemic toxicities and broad pharmacological trends where pure RA data are limited [15,16,28-30].

Discussion

Long-term low-dose GC therapy in RA sits at the intersection of proven anti-inflammatory efficacy and cumulative, dose-related toxicity. The literature reviewed here indicates that doses of ≤7.5 mg/day prednisone equivalent retain measurable clinical and structural benefits, but that these benefits are accompanied by a consistent excess of infections and a more variable, largely dose- and patient-dependent signal for skeletal and cardiovascular harm. The following sections synthesize the evidence across these domains and consider its implications for prescribing, monitoring, and withdrawal.

Efficacy and Safety Balance: Evidence from Clinical Trials

The pragmatic, double-blind, placebo-controlled GLORIA trial provides the most directly relevant randomized evidence for the population of interest, having tested add-on prednisolone 5 mg/day for two years in patients with active RA aged 65 years and older [17]. Its design deliberately gave equal weight to benefit and harm, with disease activity and radiographic progression assessed alongside a pre-specified list of adverse events of special interest [7]. Prednisolone produced a clinically meaningful reduction in disease activity and a slowing of radiographic joint damage relative to placebo, confirming that even 5 mg/day retains disease-modifying capacity when added to existing therapy [17]. Comprehensive systematic literature reviews informing international guidelines further confirm this well-established balance of therapeutic efficacy and associated safety considerations [31]. Against this, the trial demonstrated an excess of adverse events of special interest in the prednisolone arm, driven predominantly by non-severe infections rather than by serious events, fractures, or deaths [17]. Furthermore, broader randomized trial evidence comparing different bridging strategies indicates that high-dose GC regimens do not necessarily confer superior radiographic benefits over lower doses in active early disease [18].

Evidence from younger adults also helps address the limited age range of the GLORIA trial. Krause et al. [18] studied patients with active early RA and compared short-term bridging strategies using higher- and lower-dose prednisolone. This trial provides evidence about early disease control in a population not restricted to older adults, although its short-term bridging design does not establish the long-term safety of maintenance GC therapy. The 10-year analysis of early RA by Roubille et al. [24] provides additional longer-term context for severe outcomes in an early-disease population, but its observational design limits causal conclusions. Taken together, these studies broaden the evidence beyond older adults while highlighting that evidence on long-term low-dose GC safety in younger patients remains limited.

This pattern - modest but real efficacy accompanied by a narrow and largely infectious harm profile - is the central finding of the trial literature, and it frames the entire risk-benefit debate [13]. Systematic reviews and meta-analyses of randomized trials comprising over 1000 patients likewise demonstrate that continuing versus tapering low-dose GCs yields notable benefits in disease activity scores, functional ability, and radiographic progression without significantly increasing overall adverse events, except for infections [30]. Importantly, GLORIA also illustrates the limitations of the trial evidence base: a two-year horizon in an older population, in whom longer-term follow-up may be difficult, cannot capture the slowly accruing toxicities, such as osteoporotic fracture and atherosclerotic cardiovascular disease, that motivate most clinical anxiety about long-term GC use [7]. Randomized trials of low-dose GCs are generally short and underpowered for safety endpoints, which is precisely why observational data continue to dominate the harm literature despite their susceptibility to confounding by indication [12,13].

Metabolic and body-composition effects are frequently cited as a reason to avoid GCs. The GLORIA body-composition sub-study found that two years of add-on prednisolone 5 mg/day in patients aged 65 and older did not produce clinically significant changes in body weight or lean mass, though modest increases in trunk fat were observed [19]. These findings contrast with patient-reported concerns and with the broader adverse-effect profile documented by Naranjo Palacio et al. [29], who catalogued weight gain, cushingoid features, skin fragility, and hyperglycemia among the most common complaints during prolonged GC therapy. The discrepancy suggests that metabolic harm at very low doses may be subtler than commonly assumed, but the clinical significance of even modest fat redistribution in an elderly population already at elevated cardiometabolic risk should not be dismissed.

Skeletal Toxicity, Glucocorticoid-Induced Osteoporosis, and Bone Health

Bone is the organ system in which the evidence is most internally contradictory, and the evidence regarding GIOP, BMD, and fracture risk remains inconsistent across study designs. Observational data are consistently unfavorable. Cheng et al. [20] found that RA patients receiving low-dose GCs had lower BMD, a higher rate of prior fragility fracture, and a higher FRAX-estimated 10-year probability of major osteoporotic and hip fracture than RA patients not receiving GCs. In a large cohort of US patients with selected inflammatory conditions, Balasubramanian et al. [16] demonstrated a graded relationship between GC exposure and fracture risk, with risk rising as cumulative and daily dose increased. Systematic reviews and meta-analyses further corroborate these concerns, demonstrating significantly lower BMD and substantial vertebral fracture prevalence in this population [32]. Moreover, detailed clinical investigations show that BMD loss can occur even at very low thresholds, such as 2.5 mg/day, while fracture risk specifically increases at doses ≥5 mg/day [21].

Randomized and pooled trial data are less alarming. The individual participant data meta-analysis by Palmowski et al. [33], which synthesized participant-level information on BMD change and fracture over two years of low-dose GC treatment for RA, represents the methodologically strongest available assessment of this question and did not reproduce the magnitude of skeletal harm suggested by cohort studies.

Several explanations for this divergence merit discussion. First, GCs suppress the inflammatory drive that itself causes periarticular and generalized bone loss in RA, so part of their direct catabolic effect on bone may be offset by disease control [13,36]. Second, patients enrolled in trials are more likely to receive calcium, vitamin D, and bisphosphonate co-prescription, which may neutralize measurable bone loss at low doses. Furthermore, professional society guidelines emphasize systematic prevention frameworks, noting that fracture risk can increase at doses ≥2.5 mg/day (with rapid bone loss heavily concentrated in the initial three to six months) and highlighting the efficacy of anti-osteoporosis medications in preventing such damage [21,34]. Notably, Adami et al. [21] demonstrated that anti-osteoporosis treatment prevents bone loss effectively below 5 mg/day, directly supporting the argument that this risk is modifiable through early prophylaxis. Third, observational cohorts capture patients with higher disease severity, longer cumulative exposure, and greater frailty - the confounding-by-indication problem in its clearest form [16,20]. Finally, randomized trials are limited by shorter follow-up intervals and few accrued fracture events, whereas observational cohorts capture longer cumulative exposure and broader long-term real-world outcomes.

The reasonable interpretation is that low-dose GCs are not skeletally neutral, but that the magnitude of their attributable fracture risk at doses in the 5 mg/day range remains uncertain, given the inconsistency between trial and observational data; this risk may be at least partly modifiable through systematic osteoporosis prophylaxis, though confirmatory long-term evidence is lacking.

Cardiovascular Toxicity

Cardiovascular risk is the domain where dose thresholds appear most decisive. It is important to note that most of the evidence in this area derives from observational studies, which can demonstrate associations but cannot establish causation due to inherent susceptibility to confounding by indication and residual confounding. Using large administrative cohorts of RA patients on stable DMARD therapy, Coburn et al. [22] found a dose-dependent association between GC prescription and composite cardiovascular outcomes, but the association was concentrated in the older, more comorbid population and was largely absent in a younger, healthier cohort except among those with pre-existing cardiovascular risk. This suggests that baseline cardiovascular vulnerability, rather than GC exposure alone, may determine whether low-dose therapy is associated with events.

So et al. [23] reached a compatible conclusion in a population-based study specifically examining the time- and dose-dependent relationship between systemic GCs and major adverse cardiovascular events in RA, identifying a threshold effect whereby risk rose materially at higher daily doses, while very low doses were not significantly associated with excess risk. Furthermore, 10-year analyses in early RA cohorts demonstrate dose- and time-dependent risks for severe outcomes encompassing cardiovascular events, infections, and fractures [24]. Pujades-Rodriguez et al. [15] reported increased cardiovascular risk even below 5 mg/day across a large population with immune-mediated inflammatory diseases. However, this discrepancy is partly explained by population heterogeneity, as that cohort was not restricted to RA, and by the difficulty of adjusting for disease activity, which independently drives cardiovascular risk and also drives GC prescribing (a key limitation emphasized by Buttgereit & Tam [10], as well as the EULAR systematic literature review, which explicitly found cardiovascular data "inconsistent").

Regarding mortality outcomes, retrospective cohort findings indicate that doses below 5 mg/day are not significantly associated with increased mortality [25], though careful consideration of perimortal bias remains essential. Thresholds for excess all-cause mortality appear to emerge at higher exposure levels, specifically around a daily dose of 8 mg/day or a cumulative dose of 40 g.

From a mechanistic standpoint, Lend et al. [26] demonstrated in a 24-week post hoc analysis of a randomized trial population with early RA that GC treatment was independently associated with increased PCSK9 levels, a central regulator of low-density lipoprotein (LDL) recycling and hence circulating LDL cholesterol. In addition, mechanistic and clinical pathway evaluations of GC-induced diabetes emphasize that, while metabolic pathways are affected, the risk of clinically significant diabetes in RA patients is primarily concentrated at doses exceeding 10 mg/day [28]. While this provides biological plausibility for a lipid-mediated pathway by which GC exposure could contribute to atherogenesis, it should be interpreted strictly as hypothesis-generating; no study has yet linked GC-induced PCSK9 elevation to clinical cardiovascular events, and the available source is limited to a 24-week post hoc evaluation rather than long-term follow-up.

Taken together, the evidence indicates that, at very low doses, the magnitude of cardiovascular risk remains uncertain and largely derives from observational data prone to confounding by indication and the inability to fully adjust for disease activity. While risk shows a dose-dependent pattern in some observational cohorts [22] and threshold effects in others [23], it appears small and remains clinically material principally in patients who already carry elevated baseline risk.

Infectious Risk and Concomitant Immunosuppression

Infection is one of the most consistently reported complications associated with low-dose GC therapy, although the magnitude of risk varies by population and study design. George et al. [12], in a large cohort of patients with RA receiving stable DMARD therapy, found that doses of ≤5 mg/day prednisone equivalent were associated with a higher one-year incidence of hospitalized infection than no GC use. In the Medicare cohort, the estimated one-year incidence was 8.6% among patients not receiving GCs and 11.0% among those receiving ≤5 mg/day. In the Optum cohort, the corresponding estimates were 4.0% and 5.2%, respectively. Higher-dose categories were associated with progressively higher estimates in both cohorts [12]. These findings demonstrate an association, but residual confounding by disease activity, comorbidity, and treatment indication cannot be excluded.

The available evidence generally reports serious or hospitalized infections as composite outcomes rather than establishing that a specific pathogen accounts for most of the excess risk. The studies discussed here do not provide sufficient evidence to conclude that tuberculosis, hepatitis, or HIV is the predominant infection associated with low-dose GC use. Such infections remain clinically relevant in appropriate risk settings, particularly when other immunosuppressive therapies are used, but pathogen-specific screening should follow the patient’s risk profile and the recommendations relevant to the accompanying DMARD or biologic therapy. Riley and George [35] emphasized that GC-associated risk must be considered alongside the infectious risks of conventional synthetic, biologic, and targeted synthetic DMARDs.

Schenfeld et al. [1] quantified this interaction directly, demonstrating a dose-dependent association between oral GC exposure and hospitalized infectious events in RA and showing that risk was further modified by concomitant TNF inhibitor therapy.

The GLORIA trial adds nuance by showing that the randomized excess in infection at 5 mg/day was predominantly in non-severe events, without a demonstrable increase in serious infection [17]. This apparent divergence from the cohort data is best explained by statistical power and follow-up duration rather than by genuine contradiction: serious infections are uncommon enough that only very large cohorts can detect a modest relative increase [12]. The pragmatic conclusion is that patients on long-term low-dose GCs should be counseled about infection risk, kept current with vaccination, and reassessed whenever additional immunosuppression is introduced [1,35]. 

Tapering Strategies, Withdrawal, and Modern Practice Recommendations

Because cumulative exposure is central to the long-term risk-benefit balance, tapering and withdrawal are directly relevant to deciding whether maintenance low-dose GC therapy remains justified. Giollo et al. [11] examined very-low-dose GC therapy in RA and the relationship between the timing of biologic or targeted synthetic DMARD initiation and successful GC withdrawal. Earlier introduction of effective DMARD therapy was associated with greater success in discontinuation, supporting the use of GCs as a temporary bridge while steroid-sparing treatment takes effect rather than as indefinite maintenance whenever discontinuation is feasible. The SEMIRA trial [27] also demonstrated the clinical trade-off involved in tapering: among patients with RA who had low disease activity while receiving tocilizumab and prednisone 5 mg/day, tapering increased the risk of losing disease control compared with continuing prednisone. These findings support an individualized tapering strategy that considers disease activity, the effectiveness of background DMARD therapy, duration of GC exposure, and the patient’s risk of adverse effects.

Withdrawal itself carries risk. The systematic review by Naranjo Palacio et al. [29] documented withdrawal phenomena following prolonged GC therapy in RA and systemic lupus erythematosus, including symptoms attributable to hypothalamic-pituitary-adrenal (HPA) axis suppression and to disease flare. It should be noted that while HPA axis suppression is well documented at pharmacological doses, evidence for clinically significant adrenal insufficiency at doses considered physiological (≤5 mg/day prednisone) is more limited; nevertheless, case-level evidence and clinical experience suggest that even these doses can suppress the HPA axis when administered chronically, and gradual tapering remains advisable regardless of dose [29].

Buttgereit and Kvien [13], framing the controversy explicitly, argued that maintenance low-dose GC therapy remains a defensible option in selected patients, particularly where alternative therapies have failed, are contraindicated, or are inaccessible, provided the dose is minimized, the indication is periodically re-examined, and prophylaxis against predictable harm is instituted.

The evidence reviewed here identifies several patient subgroups in whom the risk-benefit balance of long-term low-dose GC therapy tilts more decisively toward harm. Older adults (≥65 years), patients with pre-existing cardiovascular disease or multiple cardiovascular risk factors, those with a history of osteoporosis or fragility fracture, individuals with prior serious infections, and patients with diabetes or pre-diabetes should be regarded as higher-risk for GC-related complications [12,20,22]. Concurrent use of biologic or targeted synthetic DMARDs, particularly TNF inhibitors, further amplifies infectious risk [1,35]. In these populations, the threshold for initiating or continuing low-dose GCs should be higher, tapering should be pursued more actively, and systematic prophylaxis, including bone-protective therapy, infection screening, vaccination, and glycemic monitoring, should be standard practice.

Limitations

Several limitations constrain the conclusions of this review. As a traditional narrative review, article identification was not exhaustive and is subject to selection bias; restriction to English-language, freely available full-text articles published within the last 10 years may have excluded relevant evidence.

The included studies were heterogeneous in design, population, comparator, GC dose, duration of exposure, and follow-up. Definitions of low-dose exposure varied, most commonly using thresholds of ≤5 mg/day or ≤7.5 mg/day prednisone equivalent, which limits the ability to identify a single dose threshold for adverse outcomes. Evidence from mixed inflammatory-disease populations was used only for context where RA-specific evidence was limited; such findings may not be directly generalizable to RA.

Most evidence on long-term harm came from observational studies, which are vulnerable to confounding by indication because GCs may be prescribed to patients with more active or refractory disease. Conversely, randomized trials generally had shorter follow-up and insufficient power to detect uncommon or delayed outcomes such as serious infection, fragility fracture, and cardiovascular events. Several key trials involved older adults, limiting generalizability to younger patients who may accumulate a longer lifetime exposure. Finally, this review used narrative synthesis and did not perform a formal risk-of-bias assessment or a new meta-analysis. The findings should therefore be interpreted as a critical summary of the available literature rather than a quantitative estimate of causal effects.

## Conclusions

Low-dose GC therapy at ≤7.5 mg/day prednisone equivalent may reduce disease activity and slow radiographic progression in RA, but these benefits must be weighed against cumulative risks. Infection is the most consistently supported safety concern, including evidence of increased hospitalized-infection risk at doses of ≤5 mg/day. The evidence for skeletal and cardiovascular harm at very low doses is less consistent and is affected by differences in study design, patient characteristics, disease activity, and cumulative exposure.

When continued GC therapy is necessary because of persistent disease activity or a lack of suitable alternatives, the minimum effective dose should be used, with regular reassessment and appropriate monitoring. Assessment and prevention of GIOP, vaccination and infection-risk review, cardiovascular risk management, and glycemic monitoring should be tailored to the individual patient. When disease control permits, gradual tapering and effective steroid-sparing DMARD therapy should be prioritized. Tapering should be individualized because disease flare and, after prolonged exposure, possible hypothalamic-pituitary-adrenal axis suppression may complicate withdrawal.
